# Elastocaloric Performance of Natural Rubber: The Role of Nanoclay Addition

**DOI:** 10.3390/molecules30143035

**Published:** 2025-07-19

**Authors:** Marica Bianchi, Luca Fambri, Mauro Bortolotti, Alessandro Pegoretti, Andrea Dorigato

**Affiliations:** Department of Industrial Engineering and INSTM Research Unit, University of Trento, via Sommarive 9, 38123 Trento, Italy; luca.fambri@unitn.it (L.F.); mauro.bortolotti@unitn.it (M.B.); alessandro.pegoretti@unitn.it (A.P.); andrea.dorigato@unitn.it (A.D.)

**Keywords:** natural rubber, elastocaloric effect, solid-state cooling, nanoclays, nanocomposites

## Abstract

This work investigates the effect of nanoclay addition—specifically natural montmorillonite (MMT) and organo-modified montmorillonite (O-MMT)—on the elastocaloric performance of natural rubber (NR), a promising material for solid-state cooling due to its non-toxicity, low cost, and ability to exhibit large adiabatic temperature changes under moderate stress (~a few MPa). Despite these advantages, the cooling efficiency of NR remains lower than that of conventional vapor-compression systems. Therefore, improving the cooling capacity of NR is essential for the development of solid-state cooling technologies competitive with existing ones. To address this, two series of NR-based nanocomposites, containing 1, 3, and 5 phr nanofiller, were prepared by melt compounding and hot pressing and characterized in terms of morphology, thermal, mechanical, and elastocaloric properties. The results highlighted that the better dispersion of the organoclays within the rubber matrix promoted not only a better mechanical behavior (in terms of stiffness and strength), but also a significantly enhanced cooling performance compared to MMT nanofilled systems. Moreover, NR/O-MMT samples demonstrated up to a ~45% increase in heat extracted per refrigeration cycle compared to the unfilled NR, with a coefficient of performance (COP) up to 3, approaching the COP of conventional vapor-compression systems, typically ranging between 3 and 6. The heat extracted per refrigeration cycle of NR/O-MMT systems resulted in approx. 16 J/cm^3^, higher with respect to the values reported in the literature for NR-based systems (ranging between 5 and 12 J/cm^3^). These findings emphasize the potential of organoclays in enhancing the refrigeration potential of NR for novel state cooling applications.

## 1. Introduction

As global average temperatures rise and heatwaves become more frequent, the use of vapor-compression air conditioners (ACs) to provide a more comfortable indoor environment has become critical for the survival of billions of people around the world [1,2,3]. Demand for air conditioners has dramatically increased in recent decades, especially in regions with severe weather conditions. According to the International Energy Agency, there were 2 billion air conditioners in use worldwide at the end of 2016, and that number is expected to rise to 5.6 billion by 2050, with China, India, and Indonesia accounting for half of the total [4]. Although ACs provide essential benefits for both physical and mental health, their environmental impact is significant, contributing to global warming, the very problem that has led to their widespread use [5]. Hydrofluorocarbons (alkyl halides), known as potent greenhouse gases, are among the most widely used refrigeration fluids. Small leaks in cooling circuits cause these fluids to be dispersed into the environment, contributing to greenhouse gas emissions [5,6]. It is therefore a matter of some urgency to break out of this vicious circle by finding alternative cooling technologies that do not involve the use of hazardous materials and are more environmentally friendly [7].

Solid-state cooling based on caloric materials is a promising and cleaner alternative to current cooling technologies. Caloric materials are solids that exhibit large entropy changes under the application (or removal) of a specific external field (i.e., an electric, magnetic, or mechanical field), and, if done adiabatically, this entropy change leads to significant temperature variations, which is the basis of solid-state refrigeration [6,7,8,9]. The resulting temperature difference (ΔT) with the applied stimulus is an indicator of the extent of the caloric effect. The larger the change in the entropy with the applied field, the greater will be the temperature change and hence the caloric effect. If the temperature variations (i.e., temperature increase when the external field is applied and temperature reduction when the external field is removed) are properly exploited, a refrigeration cycle alternative to the ones currently used may be developed. Depending on the nature of the caloric material and the external stimulus, the caloric effects can be classified as magnetocaloric, electrocaloric, elastocaloric, and barocaloric [8].

Elastocaloric materials exhibit reversible temperature changes when uniaxial tensile stress is applied and removed under adiabatic conditions [6]. Among elastocaloric materials, Natural Rubber (NR), a well-known and widely diffused bio-based material, has gained particular interest due to its elastocaloric effect being triggered by small stress values (a few MPa) and the significant temperature variations achievable (approx. 12 °C) [8,10,11,12,13,14,15,16]. Furthermore, its non-toxicity, low cost, and good fatigue properties have made NR an ideal candidate for use in solid-state cooling. The elastocaloric effect of NR is the result of two contributions. The first is the reversible orientation/disorientation of the polymer chains in the strain direction, which can be observed in most elastomers. During stretching, the orientation of the macromolecules in the stress direction induces a negative entropy variation. As a consequence, the rubber heats up and releases heat to the surrounding environment. When the material is unloaded, the return of the polymer chains to the initial high entropy state causes the cooling of the rubber, which absorbs heat from the surroundings, achieving a cooling effect. Such a relation between mechanical and thermal response is called the thermoelastic effect. The second contribution is strain-induced crystallization (SIC), i.e., partial crystallization as a result of deformation. When NR is stretched at a deformation level higher than 300% [8], the nucleation and growth of crystalline domains take place, which melts once NR returns to the unstretched state. Since crystallization and melting are exothermic and endothermic phenomena, respectively, the heat developed/released in the stretching/release phase is added to the thermoelastic heat contribution [10]. SIC is more pronounced in NR as compared to other synthetic elastomers due to the stereoregularity and limited steric hindrances of its macromolecular structure, which facilitates the alignment of NR macromolecules upon deformation [17]. The combination of thermoelasticity and significant SIC is the origin of its higher elastocaloric performances with respect to other elastomers that could be considered for this application (such as cis-butadiene rubber, chlorosulfonated polyethylene, silicon rubber and styrene-butadiene rubber [14]). In a previous work of our group, the effect of crosslinking density on the elastocaloric properties of NR was investigated. It was highlighted that a reduction of the crosslinking degree significantly enhanced the elastocaloric properties, and the least crosslinked sample achieved a coefficient of performance (COP_mat_), evaluated as the ratio between extracted thermal energy and deformational work per unit volume, equal to 2.4 [10]. Several studies have brought to light that SIC is the major contributor to the elastocaloric effect of NR [17,18,19,20]. This means that the key to enhancing its cooling performance may be to increase the capability of rubber to crystallize under strain as much as possible. The role of layered silicates, such as montmorillonite (MMT), in promoting and increasing the SIC of NR has been reported in the literature [21,22,23,24]. MMT nanofiller is characterized by a lamellar crystalline structure, in which every lamella, 1 nm thick and 200–300 nm long, presents a central octahedral sheet of alumina or magnesia alternated to two external silica tetrahedrons. These layers are stacked with a regular van der Waals gap between them. Isomorphic substitution within the lamellae generates negative charges that are generally counterbalanced by Na^+^ or Ca^2+^ cations located in the interlayer region [25,26,27]. As the stacks are held together by relatively weak bonds, the intercalation of small molecules between the layers can be performed to obtain a more organophilic clay. The hydrated cations of the interlayer can be exchanged with cationic surfactants such as alkylammonium or alkylphosphonium salts, generating thus organo-modified (OM) clays [28,29]. Carretero-Gonzales et al. found that the addition of 15 phr of nanoclays (both natural sodium MMT and organically modified MMT) in crosslinked NR provided a more homogeneously distributed rubber network structure and induced an early onset as well as enhancement of crystallization under uniaxial deformation. The onset of SIC moved from 300% strain in the unfilled rubber to 120–140% strain in the filled NR [21,22]. In addition, they found that the strong orientation of the nanoclays along the stretching direction favored a further orientation of the macromolecules, promoting the nucleation of crystalline domains under stretching. Nie et al. also observed that nanoclays (0.5 phr in the rubber formulation) changed the SIC behavior of NR, as the crystallinity of the nanoclay-filled NR was higher than that of the unfilled NR at the same strain level. This highlights that the addition of nanoclays accelerated the strain-induced crystallization rate [23]. The research of Qu et al. revealed that the maximum degree of SIC of organo-modified MMT-filled NR was achieved for a nanoclay content equal to 5 wt%. At higher filler contents, the crystallinity under strain was reduced, probably due to a lower dispersion and exfoliation of OM nanoclays in the rubber [24].

However, while several studies have examined the role of nanoclays in promoting SIC, to the best of the authors’ knowledge, their direct impact on the elastocaloric performance of NR has not yet been investigated. This represents a critical gap, as an increase in SIC could directly translate to enhanced cooling efficiency and higher COP. Therefore, the focus of this work is to explore for the first time whether the increased SIC promoted by nanoclays in NR could have a positive impact on its elastocaloric properties. Both natural and organo-modified MMT were thus melt compounded in NR at different concentrations (from 1 up to 5 phr), and the elastocaloric properties of the resulting nanocomposites were correlated to their morphological and thermo-mechanical behavior.

## 2. Results and Discussion

### 2.1. Evaluation of the Density and Crosslinking Degree

In Table 1, the density and crosslinking density values of unfilled NR and the relative nanocomposites are reported.

The addition of nanoclays to NR promotes a slight density increase, which is more pronounced in the case of natural sodium MMT. This result is reasonable since the density of the nanoclays, reported in Table 7, is higher than that of NR. As regards the crosslinking density, it is interesting to notice that the introduction of the organoclays induces a higher crosslinking degree. For instance, moving from unfilled NR to NR_3O-MMT, the crosslinking density increases from 2.94 mol·10^−4^/cm^3^ to 3.73 mol·10^−4^/cm^3^. This experimental evidence has been reported in the literature and can be attributed to the amine groups present in the O-MMT structure, which come from the organophilization of the nanoclays. The amine group can act as a vulcanizing agent and thus promote an increase in the crosslinking degree [30]. In our previous work [10], it was demonstrated that with increasing degree of crosslinking (in a range of crosslinking density comprised between 2.94 and 5.24 mol·10^−4^/cm^3^), the elastocaloric properties decrease. This aspect has been taken into account and will be considered in the discussion of the elastocaloric properties (see Section 2.5).

### 2.2. Structural and Morphological Analysis

**Figure 1:**
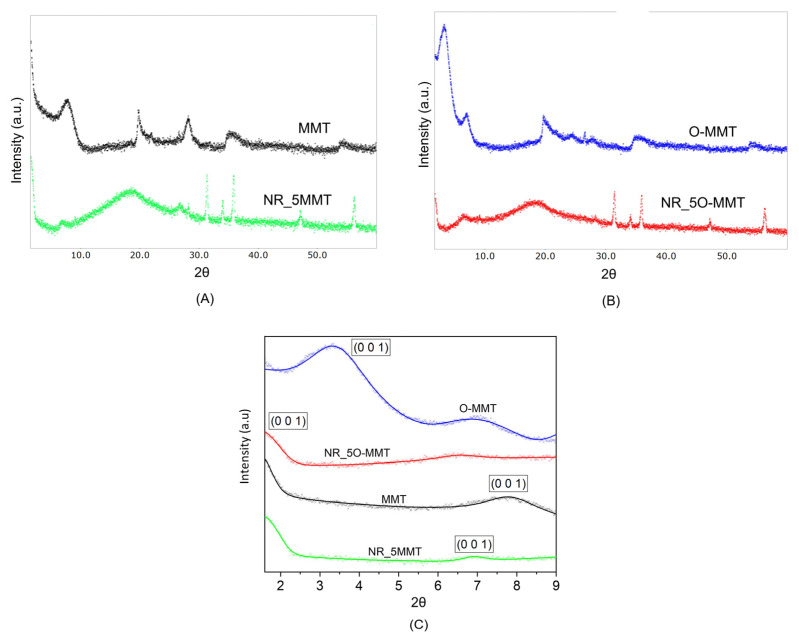
Results of XRD diffraction analysis on neat nanoclay and the relative nanocomposites (nanoclay loading = 5 phr). High resolution XRD patterns of (**A**) neat MMT and NR_5MMT, (**B**) neat O-MMT and NR_5O-MMT samples, and (**C**) experimental fit of the basal peak performed with a Pseudo-Voigt function.

The X-ray diffraction (XRD) patterns obtained on neat nanoclays, NR_5MMT and NR_5-O-MMT nanocomposites are reported in Figure 1A,B, while Figure 1C shows the experimental fit of the basal peak, obtained by fitting the XRD patterns with a Pseudo-Voigt function without considering a structural model for simplicity.

The signal of the basal peak of neat natural nanoclay is localized at 2θ = 8° and slightly shifts to a lower 2θ value in the NR_5MMT sample. As regards the organoclays, the (001) reflection peak, observed at 2θ = 3°, significantly shifts toward lower 2θ values in the NR_5O-MMT nanocomposite. However, due to a probable overlap with the direct beam signal, it is difficult to detect it clearly. Given these challenges, the basal peak position for O-MMT in the NR_5O-MMT was assumed at 2θ = 1.6°, at the edge of the measurement range. Nonetheless, the true peak could lie at even lower 2θ values or might be absent in the case of complete organoclay exfoliation. The d_001_ spacing values calculated using Bragg’s law are reported in Table 2.

As shown in Table 2, the interlamellar distances evaluated for the neat nanoclays are in good accordance with the values reported on the manufacturer’s data sheets (see Table 7). The interlayer spacing of the natural nanoclays slightly increases from 11.40 Å (for the neat MMT) to 12.68 Å in the NR_5MMT sample (i.e., an increase of 11.2%). The strong interlamellar interactions and the small initial d_001_ spacing of MMT result in very limited intercalation in the NR matrix [26]. The situation is different for the organoclays. The shared hydrophobic character of O-MMT and the NR matrix enhances their mutual compatibility, thereby promoting a strong intercalation (or exfoliation) of the organoclays, with d_001_ spacing passing from 26.08 Å up to values higher than 51.70 Å.

**Figure 2:**
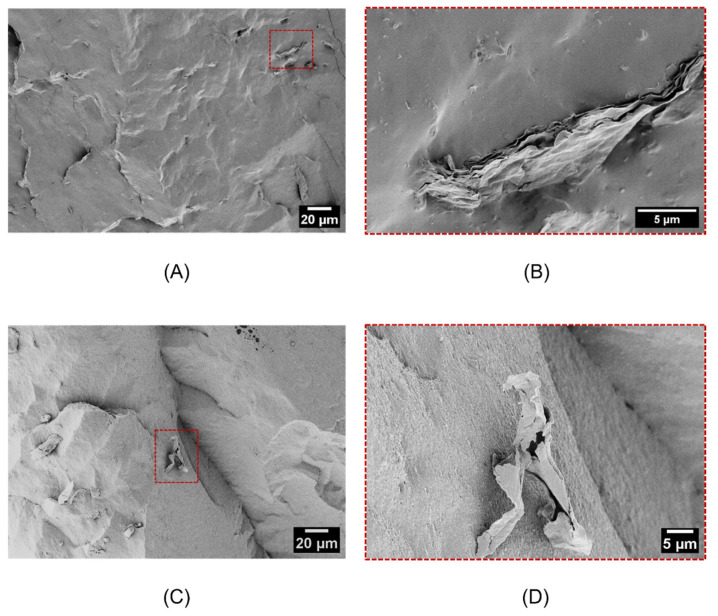
FESEM micrographs at different magnifications of the cryofracture surface of the produced NR-based nanocomposites. (**A**,**B**) NR_5MMT, (**C**,**D**) NR_5-O-MMT.

Scanning electron microscope (SEM) observations were also performed on the NR nanocomposites to investigate the nanoclay dispersion within the NR matrix. Representative FESEM micrographs of the cryofracture surface of NR_5MMT and NR_5O-MMT samples are presented in Figure 2A–D. In particular, Figure 2A,B shows the morphological features of the NR_5MMT sample, while Figure 2C, D shows the morphological features of the NR_5O-MMT nanocomposite.

The organization of the nanoclay platelets in the rubber matrix plays a crucial role in their reinforcing effect. From Figure 2A–D, it is possible to appreciate that the use of natural sodium MMT leads to the production of a poorly intercalated nanocomposite. Indeed, the platelets are densely stacked and aggregated, indicating a poor affinity with the rubber matrix [31]. On the contrary, SEM micrographs of the NR_5OMMT sample reveal isolated nanoclay platelets in the matrix, suggesting the exfoliation of the organoclays and a good affinity with NR [31]. This outcome aligns with the results of the XRD analysis and suggests that in O-MMT nanocomposites the nanoclays could be strongly intercalated and/or partially exfoliated. The hydrophilic nature of MMT accounts for its poor compatibility with the hydrophobic NR matrix. Conversely, the shared hydrophobic character of O-MMT and NR enhances their mutual compatibility, thereby promoting a finer nanofiller dispersion.

### 2.3. Thermal Properties

The specific heat capacity, thermal diffusivity, and thermal conductivity values of the unfilled NR and the relative nanocomposites, obtained from Laser Flash Analysis (LFA), are reported in Table 3.

Regardless of the type and amount of nanoclays, the prepared nanocomposites do not show significant differences in thermal properties. Compared to the unfilled NR, the nanofilled samples exhibit a very slight increase in specific heat capacity and thermal conductivity. This is probably due to the presence of nanoclays, which are characterized by a higher thermal conductivity (~0.7 W/m·K) with respect to the NR matrix [32]. Since the thermal properties of the NR nanocomposites are comparable, any difference in their elastocaloric performances among the samples can not be attributed to their different thermal behavior. Also, differential scanning calorimetry (DSC) analysis, not shown in this work for brevity, did not reveal differences in the thermal transitions (T_g_) of the produced nanocomposites.

### 2.4. Mechanical Properties

**Figure 3:**
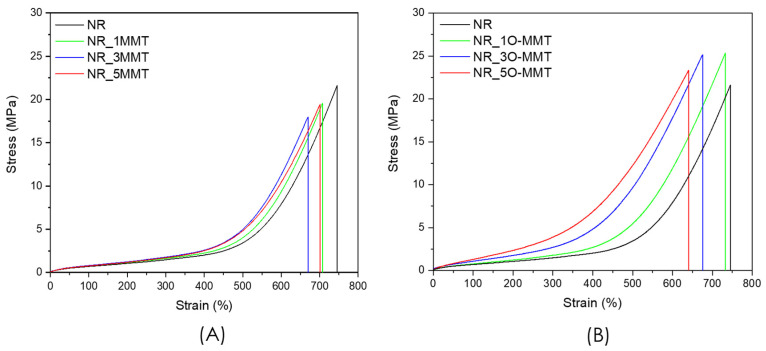
Representative stress–strain curves from quasi-static tensile tests on (**A**) NR and NR/MMT nanocomposites, (**B**) NR and NR/O-MMT nanocomposites.

Figure 3A,B shows the stress–strain behavior of the unfilled NR and the relative nanocomposites. Specifically, the representative stress–strain curves of NR/MMT samples are reported in Figure 3A, while in Figure 3B the stress–strain plots of NR/O-MMT samples are shown. The most important results of the quasi-static tensile tests are reported in Table 4.

By comparing Figure 3A and Figure 3B, the effect of nanoclay type and content on the mechanical properties of the nanocomposites is evident. Focusing at first on the mechanical behavior of NR/MMT samples, the stress–strain curves of the nanocomposites overlap with that of the unfilled NR up to a strain of approximately 300%. This observation is also confirmed by the E_100–200_ and σ_200%_ values reported in Table 4, which show no significant difference among these samples. At strain levels above 300%, the stress–strain curves show a slight stiffening in the NR/MMT nanocomposites with respect to neat NR. For example, NR_5MMT exhibits a σ_400%_ value of 2.56 MPa, +22% compared to neat NR. Overall, the addition of MMT has promoted a slight decrease in strain and stress at break, and specific tensile energy to break. The most significant reduction in E_b_ is registered in NR_1MMT (−11% compared to neat NR), while for σ_b_ in NR_5MMT (−11% compared to neat NR). Given the obtained results and the considerations drawn from the morphological analysis, it can be concluded that the poor intercalation of the rubber into the nanoclay galleries and the limited filler/NR interaction (due to the hydrophilic nature of the natural nanoclays and the hydrophobic nature of the NR matrix) have caused a limited reinforcement. A different behavior is observed in the case of NR/O-MMT samples. At the same filler loading, a stronger reinforcing effect is achieved compared to NR/MMT nanocomposites. The mechanical response of the nanocomposites diverges from that of neat NR from the outset, with increases in the elastic modulus as well as in σ_200%_, and σ_400%_ as the nanoclay content increases. For example, NR_5O-MMT exhibits an elastic modulus of 0.77 MPa and a σ_400%_ of 5.36 MPa (+120% and +155% with respect to neat NR, respectively). The tensile strength of NR improves with the addition of 1 and 3 phr of O-MMT, whereas at 5 phr it becomes comparable to that of unfilled NR, likely due to aggregation phenomena occurring at filler contents above 3 phr. A similar trend is observed for the specific energy to break. The improved mechanical properties with respect to MMT-filled nanocomposites can be explained on the basis of XRD and SEM analysis, which showed the intercalation/exfoliation of O-MMT in the NR matrix. The higher crosslinking density found in O-MMT/NR samples with respect to the neat NR (see Table 1) could also be in part responsible for the stiffening of these nanocomposites, and also for the slight σ_b_ decrease observed at elevated O-MMT amounts. In agreement with the results of quasi-static tests, the Shore A hardness also undergoes an increase upon nanoclay addition, proportional to the nanofiller concentration and more pronounced in the case of O-MMT.

For the evaluation of the elastocaloric effect, the samples were deformed to 400% strain, as our previous study demonstrated that this deformation level guaranteed the best cooling performance [10]. As reported in Table 4, the stress required to reach this level of deformation in all the NR nanocomposite samples (σ_400%_) is below 6 MPa. Considering that the stress required to compress the refrigerant fluids in current air conditioners is on the order of 1 MPa, it can be concluded that the stress levels involved in exploiting the elastocaloric properties of NR are of a comparable order of magnitude.

**Figure 4:**
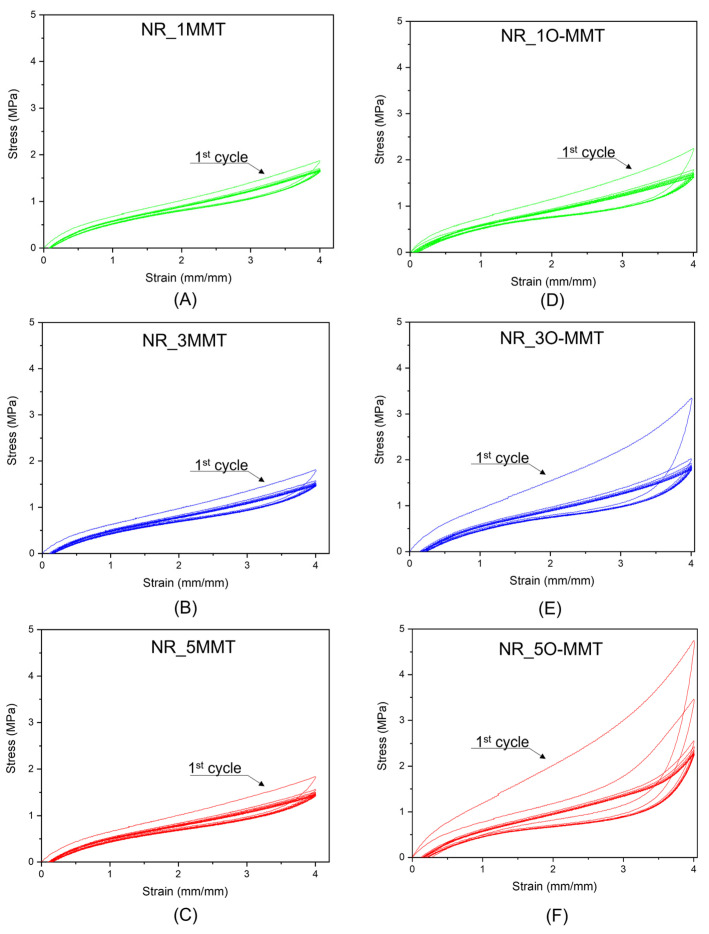
Stress–strain curves of the NR-based nanocomposites subjected to uniaxial cyclic tension (maximum strain level = 400%). (**A**) NR_1MMT, (**B**) NR_3MMT, (**C**) NR_5MMT, (**D**) NR_1O-MMT, (**E**) NR_3O-MMT, (**F**) NR_5O-MMT. The first cycle is evidenced in all the graphs.

The use of NR in a solid-state cooling device has one key implication: NR must withstand a high number of loading–unloading cycles while maintaining consistent mechanical performance. Given the susceptibility of NR composites to the Mullins effect, it is of fundamental importance to explore the mechanical response of the produced samples under cyclic deformation. Figure 4A–F shows the stress–strain response of the NR nanocomposites under cyclic tensile loading when a maximum strain level of 400% is applied. In particular, Figure 4A–C illustrates the mechanical behavior of NR/MMT samples, while Figure 4D–F illustrates that of NR/O-MMT nanocomposites.

**Figure 5:**
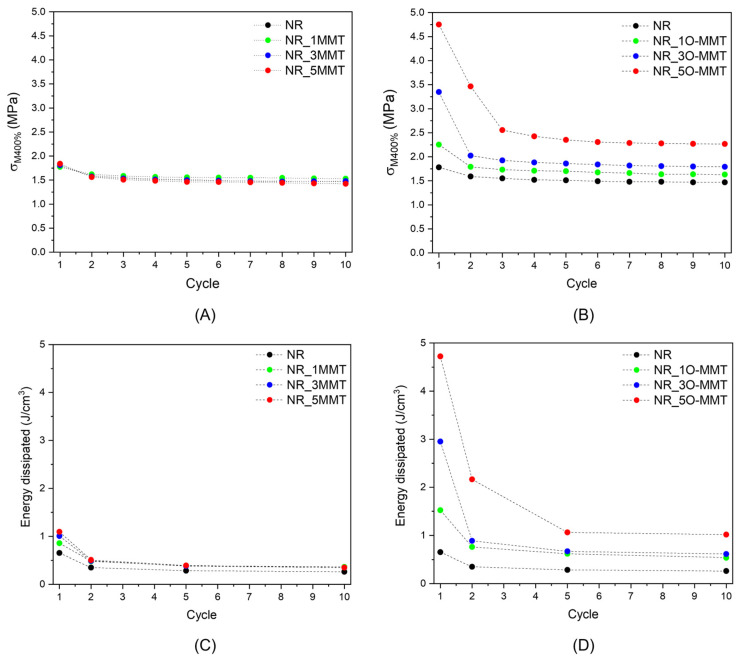
Results from the uniaxial cyclic tension tests on the neat NR and the relative nanocomposites. (**A**,**B**) Maximum stress at 400% and (**C**,**D**) specific energy dissipated as a function of cycle number. (**A**,**C**) refer to NR/MMT samples, while (**B**,**D**) refer to NR/O-MMT samples.

The softening of the NR nanocomposites, as well as the hysteresis phenomenon, is well visible in all the samples. However, when comparing the mechanical behavior of the two series of NR nanocomposites, several considerations emerge. Focusing first on NR/MMT samples, it is possible to notice that after the first loading–unloading cycle, the mechanical response of the nanocomposites tends to stabilize. In addition, the filler content seems to have only a slight influence on the Mullins effect. Comparing the behavior of the NR/O-MMT samples with that of the MMT nanofilled ones, it can be noticed that the Mullins effect is more pronounced, given the higher hysteresis of the first loading–unloading cycle and the drop in peak stress (i.e., the stress at 400% strain) moving from the first to the subsequent cycles. Additionally, by increasing the filler content in the NR matrix, the softening of the nanocomposites is more evident. Moreover, at elevated O-MMT amounts, more than one cycle is needed to stabilize the mechanical response of the samples. To enable a more accurate comparison of the cyclic mechanical behavior of the nanocomposites, the peak stress and the energy dissipated during the 1st, 2nd, 5th, and 10th cycles have been calculated and shown in Figure 5A–D.

As it has already been appreciated from Figure 4A–F, the softening of NR/O-MMT samples is more pronounced than that shown by NR/MMT nanocomposites (Figure 5A,B), and the same can be said for the energy dissipated (Figure 5C,D). For example, NR_5O-MMT exhibits an energy dissipation of 4.8 J/cm^3^ in the first loading–unloading cycle, about five times greater than that determined for the NR_5MMT sample (1.1 J/cm^3^). At least three cycles seem necessary to stabilize the mechanical response of O-MMT nanocomposites, whereas two cycles seem sufficient for the MMT nanofilled once. Up to now, the origin of the Mullins effect at the microscopic scale is unclear. Among the theories advanced to explain this effect, there are some regarding molecules slipping [33]. Particularly, some authors have proposed that, during the first extension, the macromolecules slip over the filler surface and new physical bonds are instantaneously created along the chains. The new bonds would be of the same physical nature as the original ones but would appear at different places along the polymeric chain, causing the softening of rubber and marked hysteresis phenomena. Considering this, the different behavior found in the two series of nanocomposites can be attributed again to the different dispersion and interactions the fillers have with the NR matrix. The hydrophilic nature of MMT causes this filler to weakly interact with the NR chains, and, consequently, the nanocomposites exhibit a Mullins effect comparable to the unfilled rubber. Instead, the hydrophobic tails of O-MMT allow a more intense interaction with the NR matrix, promoting the formation of a higher density of physical bonds between nanoclays and NR macromolecules. In addition, the elevated dispersion (or exfoliation) of O-MMT within the NR observed in the SEM micrographs (Figure 2C,D) greatly increases the surface area of interaction between the clays and the rubber. The rapture and re-formation of the O-MMT-rubber physical bonds during the first cycle could explain the greater Mullins effect observed in organoclay-filled nanocomposites.

In summary, these findings emphasize the importance of pre-stretching the produced nanocomposites immediately before their application, particularly for the higher filler loading samples. However, the Mullins effect is a reversible process and, if the rubber is left to rest for prolonged periods, a partial recovery of this effect may occur. This means that NR-based nanocomposites in solid-state cooling devices will likely require a pre-stretching phase before application. Future research will aim to explore this aspect further, given its significant practical implications.

**Figure 6:**
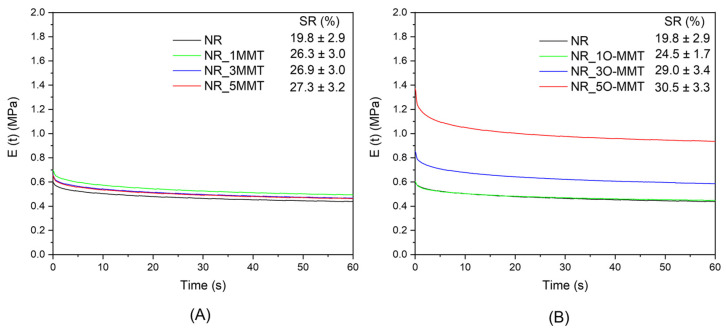
Results from stress relaxation tests on the neat NR and the relative nanocomposites. Relaxation modulus as a function of time of (**A**) NR/MMT samples and (**B**) NR/O-MMT samples.

When rubber is maintained in the stretched state for a certain time interval, stress relaxation phenomena occur. The process of stress relaxation takes place by reversible changes caused by the reorientation of the molecular network, breakage and re-formation of entanglements, and breaking of physical bonds [34]. The relaxation modulus as a function of time of the neat NR and the relative nanocomposites is shown in Figure 6A,B. In the graphs, the percentage of stress relaxation after 60 s is reported. To mitigate the influence of the Mullins effect, the samples were stretched three times before investigating their stress relaxation behavior.

In accordance with the results of quasi-static tests, NR/O-MMT samples exhibit a higher relaxation modulus with respect to MMT-filled nanocomposites, and it increases with filler content. Such a trend is not observed in the MMT nanocomposites, for which the stiffening effect promoted by the addition of nanoclays is limited. Once again, these results can be explained considering the better dispersion of organoclay lamellae and their stronger interaction with the NR matrix. The stress relaxation is more pronounced in NR-filled nanocomposites compared to unfilled NR, in agreement with findings reported in the literature [35]. For both nanocomposite series, SR increases with filler content, and, for a nanoclay loading of 3 and 5 phr, NR/O_MMT samples show slightly higher SR values than NR/MMT samples. Again, these results may be attributed to the stronger filler–polymer interaction in the case of O-MMT. While such interactions improve the mechanical performance, they also contribute to slightly greater stress relaxation. Indeed, the increased number of interactions promoted by the nanoclay exfoliation results in more frequent breakage of physical filler–polymer bonds during the relaxation process. Overall, the stress relaxation behavior of the two series of nanocomposites is quite comparable, and the calculated SR values could be considered compatible with a solid-state cooling device. However, it will be possible to have a clear idea of the practical implications of the obtained SR values only once a prototype is developed.

### 2.5. Evaluation of the Elastocaloric Effect

**Figure 7:**
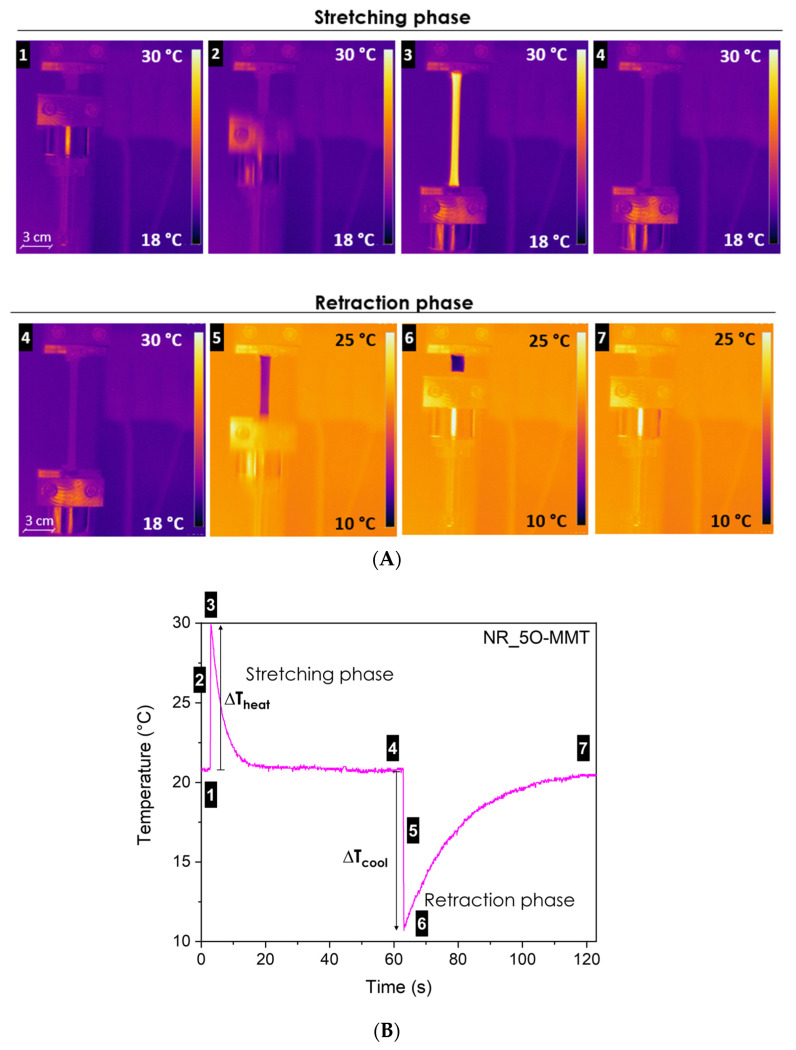
Example of the temperature variations in the NR_5O-MMT sample during the stretching and retraction phase. (**A**) Images taken from the thermal camera during the elastocaloric test and (**B**) temperature variation as a function of time.

**Figure 8:**
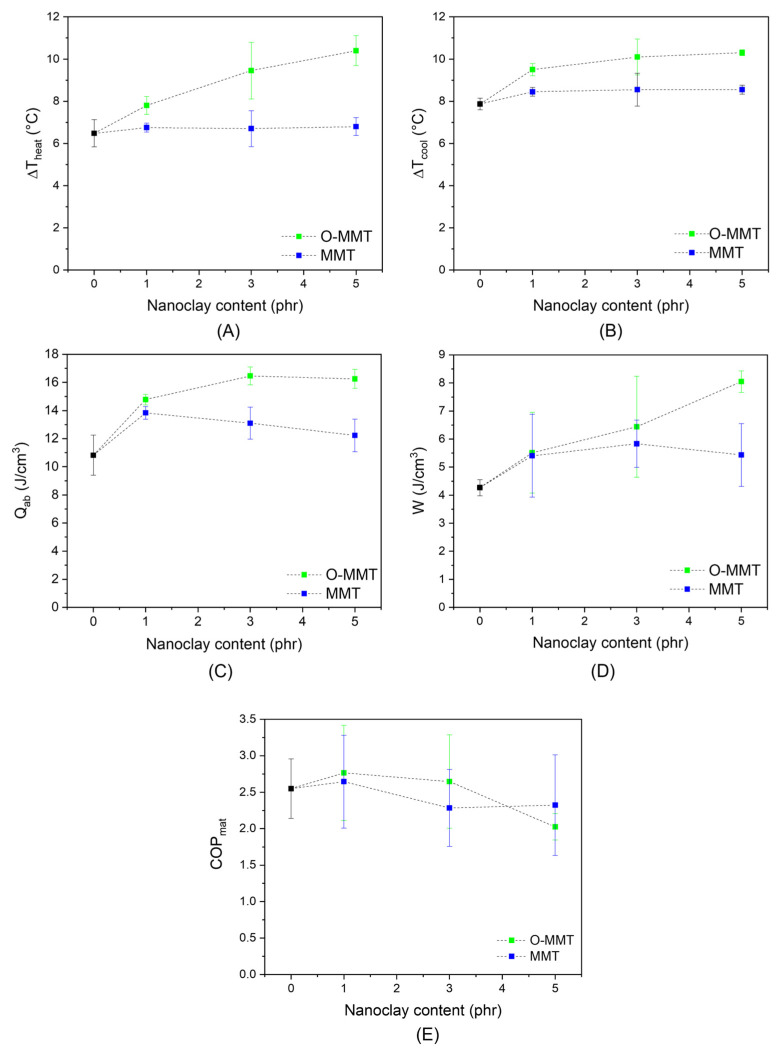
Results of the elastocaloric tests on the neat NR and the relative nanocomposites. (**A**) Maximum ∆T_heat_, (**B**) maximum ∆T_cool_, (**C**) heat extracted from the environment per unit volume of material (Q_ab_,), (**D**) specific work of deformation per unit volume of material (W), (**E**) coefficient of performance (COP_mat_).

Figure 7A,B shows representative images of the temperature variation as a function of time of the NR-5O-MMT sample, both in the stretching and retraction phase, while in Figure 8A–E, the most important results of the elastocaloric tests on the neat NR and the relative nanocomposites are reported.

Figure 7A,B shows in a graphical way the elastocaloric effect observed in the NR_5O-MMT sample. It is possible to notice that the adiabatic stretching of the sample induces a positive temperature variation, ∆T_heat_, caused by both the thermoelastic effect and the strain-induced crystallization. Maintaining a constant deformation level (400%), the sample temperature drops until reaching thermal equilibrium. When the sample is rapidly unloaded, a negative temperature variation is registered, ∆T_cool_, caused by the melting of the crystallites developed during the stretching phase and the thermoelastic effect. A similar trend has been observed in all the other nanocomposites, for which thermograms have not been reported for brevity.

The elastocaloric properties of the unfilled NR and the relative nanocomposites are reported in Figure 8A–E. Two main considerations emerge from the analysis of these results. The type of nanoclay influences not only the mechanical properties of the nanocomposites, as it has been previously observed, but also their elastocaloric performance. Higher values of ∆T_heat_ and ∆T_cool_ have been registered in O-MMT nanocomposites with respect to unfilled NR and MMT/NR samples, especially at elevated organoclay amounts. For example, NR_5O-MMT shows a ∆T_heat_ of 10.4 °C and a ∆T_cool_ of 10.3 °C, +63% and +32% than neat NR, respectively. On the other hand, natural sodium nanoclay seems to have a very limited positive effect on the cooling capacity of NR. By comparing ∆T_heat_ and ∆T_cool_ values, it can be appreciated that, for both the unfilled rubber and NR/MMT samples, the absolute values of ∆T_cool_ are slightly greater than those of ∆T_heat_, while for the NR/O-MMT nanocomposites, the temperature changes recorded during stretching and retraction phases are similar. This can be explained by examining the mechanisms contributing to the rubber temperature change. The mechanisms involved and their role in the temperature variations are summarized in Table 5. The up arrows indicate that the mechanism promotes the heating of the sample, while the down arrows indicate that the mechanism promotes the cooling of the rubber.

It is important to highlight that internal friction between NR macromolecules and nanoclay-macromolecules contributes to the temperature variation in NR, but it is not a mechanism associated with the elastocaloric effect. The elastocaloric effect arises from reversible processes, such as strain-induced crystallization (SIC) and the thermoelastic effect. In contrast, internal friction is a dissipative phenomenon that generates heat both during the loading and the unloading stages. During the stretching phase, the temperature increase, ∆T_heat_, results from the combined effects of the thermoelastic response, SIC, and macromolecular friction. Among these mechanisms, SIC evolves while the samples are maintained in the stretched state, with the greatest part of crystallites developing in the first milliseconds after the stretching [20]. Consequently, only a part of the heat generated during their formation contributes to ΔT_heat_. When the sample is unloaded, the melting of the crystallites and the thermoelastic effect positively contribute to the ∆T_cool_, while the frictional heat is a mechanism that plays havoc with the cooling of the rubber, reducing ∆T_cool_ and the cooling effect. If the friction is almost negligible, it is reasonable that ∆T_cool_ is higher than ΔT_heat_ since, during unloading, all crystalline domains, regardless of their formation time, melt, thus fully contributing to ΔT_cool_. If internal fiction is not negligible, ΔT_cool_ lower than or equal to ∆T_heat_ is expected. The higher contribution of internal friction found in NR/O-MMT samples with respect to MMT nanocomposites is further evidence of the better dispersion of O-MMT within the NR matrix.

The heat extracted from the environment during the retraction phase, Q_ab_, is the parameter that quantifies the cooling capacity of caloric materials. As shown in Figure 8C, the capability of NR/O-MMT samples to absorb thermal energy from the environment is greater than unfilled NR and NR/MMT nanocomposites. The difference between the two series of NR nanocomposites is more marked for a filler content equal to 3 and 5 phr. In one single loading–unloading cycle, NR_3O-MMT and NR_5O-MMT samples result in being capable of extracting more than 16 J/cm^3^ of heat from the surrounding environment, compared to 11 J/cm^3^ of the unfilled NR (i.e., an increase of 45%). According to the literature, the heat extracted per refrigeration cycle of NR-based systems ranges between 5–12 J/cm^3^ [14,17], lower than the values obtained in this work. These findings highlight that the selection of a proper nanofiller is fundamental for enhancing the elastocaloric properties of NR. In particular, the affinity and compatibility between the rubber and the filler play a key role in the elastocaloric performances. As reported in Table 1, the crosslinking density of NR/O-MMT nanocomposites was found to be higher compared to unfilled NR and NR/MMT samples. In our previous work, it was demonstrated that an increased degree of crosslinking leads to a decrease in the cooling capacity of NR [10]. This suggests that the presence of O-MMT in the NR matrix largely overcompensates for the negative impact caused by the higher crosslinking density.

Some hypotheses have been advanced to explain the enhanced cooling capacity observed in the NR/O-MMT samples. First, the presence of organo-modified nanoclays could facilitate a greater degree of strain-induced crystallization (SIC) in NR, owing to the O-MMT exfoliation and the improved polymer–filler interactions that promote molecular alignment under deformation. Second, the O-MMT could contribute to a more homogeneous rubber network structure, as reported in the literature [21]. A more uniform macromolecular structure, characterized by a more consistent chain length and crosslinking density, allows for greater conformational changes during stretching, resulting in a larger entropy change between the undeformed and stretched states [36]. Finally, the observed improvement may also stem from a strain amplification effect induced by the nanoclays, promoting a higher thermoelastic effect. Further investigation will be necessary to validate all these hypotheses.

Although organoclay-filled NR samples exhibit higher Q_ab_, no significant differences are observed in COP_mat_ (Figure 8E) between the two series of NR nanocomposites, and the COP_mat_ is found to range between 2 and 3. This is attributed to the greater work of deformation required for the NR/O-MMT samples (Figure 8D) with respect to MMT nanocomposites. For example, the work of deformation of NR_5-O-MMT is 8.0 J/cm^3^, compared to 4.3 J/cm^3^ of neat NR and 5.4 J/cm^3^ of the NR_5MMT sample. The higher W values observed in O-MMT nanocomposites are due to the stiffening effect provided by the better nanofiller dispersion and the higher polymer/filler interaction.

In this work, the heat exchange between the rubber and the surrounding environment occurred by forced convection using a fan positioned at a distance of 50 cm from the samples. The choice to work in a forced convection regime was dictated by the need to reduce the refrigeration cycle time (stretching + retraction phase), necessary to enhance the cooling power of a solid-state AC. In Table 6, the characteristic times of temperature dampening (τ_c_) evaluated during the stretching and retraction phases are reported.

The characteristic times of temperature dampening are more than halved in the condition of forced convection. Keeping the time of the stretching and retraction phases constant, this choice has allowed us to reduce the duration of a refrigeration cycle from 8 min, as reported in our previous work [10], to 2 min. The τ_c,retraction_ calculated in the retraction phase, remains higher than τ_c,stretching_, also in conditions of forced convection. Moreover, during stretching, the specimen becomes thinner, further favoring the heat transfer. In conclusion, the obtained results emphasize the potential of organoclays in optimizing the elastocaloric behavior of NR and open new avenues for exploring the effect of other nanofillers on its elastocaloric performance.

## 3. Materials and Methods

### 3.1. Materials

Technically Specified Rubber (TSR) grade SMR 10 (Standard Malaysian Rubber), kindly supplied by Marangoni S.p.a. (Rovereto, TN, Italy), was used as the natural rubber matrix for this work. The chemicals, i.e., zinc oxide (curing activator), sulfur (vulcanizing agent), and stearic acid (curing activator), were provided by Rhein Chemie (Cologne, Germany). The zinc dibutyldithiocarbamate (ZDBC) accelerator was acquired from Vibiplast srl (Castano Prino, MI, Italy). Natural montmorillonite (Cloisite^®^ Na^+^) was provided by Southern Clay Products, Inc. (Gonzales, TX, USA), while organo-modified montmorillonite (Cloisite^®^ 20A) was obtained from BYK Additives and Instruments (Wersel, Germany). The main characteristics of the selected clays are summarized in Table 7.

To evaluate the crosslinking density and the density of the NR samples, methanol (density: 0.791–0.793 g/cm^3^ [38]), acetone (density: 0.788–0.792 g/cm^3^ [39]) and toluene (density: 0.862–0.872 g/cm^3^ [40]) obtained from Carlo Erba Reagents S.r.l (Cornaredo, MI, Italy), were utilized. All the materials were used as received.

### 3.2. Sample Preparation

The formulation in parts per hundred rubber (phr) of the prepared NR nanocomposites is reported in Table 8. The nomenclature chosen for the NR nanocomposites is constituted by the term NR followed by a number, indicating the nanoclay content in phr in the formulation, and the term MMT or O-MMT, to indicate the type of nanoclay. The vulcanizing system was chosen on the basis of our previous study [10]. The nanoclay content (1, 3, and 5 phr) was selected to favor SIC while avoiding a pronounced stiffening effect.

The rubber compounds were prepared in accordance with ASTM 3182 standard, following the optimized process parameters reported in our previous paper [10]. Initially, NR was masticated for 5 min in an internal mixer (Thermo Haake Rheomix 600, Thermo Fisher Scientific, Waltham, MA, USA), equipped with counter-rotating rotors, and operating at 50 °C and 60 rpm. The nanoclays, pre-dried in an oven at 60 °C for 48 h, were subsequently added and mixed with the NR for 2 min. Zinc oxide, stearic acid, ZDBC, and sulfur were then introduced, and the resulting compound was further mixed and homogenized for two minutes. Vulcanization was then carried out in a Carver hydraulic press at a temperature of 120 °C and a pressure of 3 bar. The dimensions of the produced NR nanocomposites were 120 × 120 × 1 mm^3^.

### 3.3. Characterization

#### 3.3.1. Evaluation of the Density and Crosslinking Degree

The density of the NR nanocomposites was measured by using Archimedes’ principle, following the standard ASTM D792. The tests were performed at room temperature using the Mettler ME-33340 kit (Mettler Toledo, Columbus, OH, USA). The specimens were weighed in air and immersed in methanol. The density was calculated using Equation (1):

(1) $$\rho =\frac{a}{a-b}\times M\;$$

where *a* is the weight of the material in air, *b* is the weight of the sample immersed in methanol, and *M* is the density of methanol at the testing temperature (0.791 g/cm^3^ determined by using a Mettler kit ME-33340). Three specimens were tested for each composition.

To evaluate if the nanoclay addition affected the NR macromolecular structure, the crosslinking density (*υ*) of nanocomposites was determined by equilibrium swelling measurements in toluene, according to ASTM D 6814 standard. Initially, the acetone-soluble fraction in the NR was removed by immersing the samples in acetone for 3 days and subsequently drying them in a ventilated oven at 60 °C for 24 h. The specimens were then immersed in toluene for 4 days until the equilibrium state was achieved. After this period, the specimens were taken out, the swollen mass was weighed (*w_s_*) and subjected to drying to eliminate excess solvent in a ventilated oven at 60 °C. Finally, their dried mass was recorded (*w_d_*). The crosslinking density (*υ*) was calculated through Equation (2), reported by Flory-Rehner [41]:

(2) $$\upsilon =\frac{-\ln\left(1-V_{r}\right)+V_{r}+ΧxV_{r}^{2}}{V_{1}x(V_{r}^{\frac{1}{3}}-V_{r})/2}$$

where *Χ* is the Flory–Huggins polymer–solvent interaction parameter and *V*_1_ is the molecular volume of solvent, taken as 106.288 cm^3^ mol^−1^ and 0.391, respectively. *V_r_* is the volume fraction of polymer in a swollen network in equilibrium with pure solvent, calculated according to ASTM D6814 standard, as reported in Equation (3):

(3) $$V_{r}=\frac{\frac{w_{d}}{\rho_{d}}}{\frac{w_{d}}{\rho_{d}}+\frac{w_{so}}{\rho_{s}}}$$

where *w_d_* is the weight of dry rubber, *ρ_d_* is the density of dry rubber, *w_so_* is the solvent absorbed by the sample, evaluated as the difference between *w_s_* and *w_d_*, and *ρ_s_* is the density of toluene (0.867 g/cm^3^). The density of dry natural rubber was determined by Archimedes’ principle in methanol according to Equation (1). Four specimens were tested for each NR formulation.

#### 3.3.2. Structural and Morphological Analysis

X-Ray diffraction data were taken with a Rigaku D/Max-B X-ray diffractometer (Rigaku Holdings Corporation, Akishima, Tokyo, Japan) in Bragg–Brentano parafocusing geometry, a diffracted beam monochromator, and a conventional Cu target X-ray tube set, to 40 kV and 30 mA. Acquisition range (expressed in terms of 2Θ angle) was set from 1° up to 60°, with a 2Θ step of 0.02° and an acquisition time of 2 s per step. XRD measurements were performed on specimens having dimensions 25 × 25 × 1 mm^3^, and only one specimen was tested for each composition.

The nanoclays dispersion in the NR matrix was investigated using a field emission scanning electron microscope (FESEM) AG-SUPRA40 (Carl Zeiss, Oberkochen, Germany), operating at an acceleration voltage of 2.5 kV. The samples were soaked in liquid nitrogen for 1 h and then cryofractured. The fracture surface was analyzed after Pd-Pt 80/20 sputtering to provide enhanced electrical conductivity. Secondary electrons were used to image the cryofracture surfaces. Specimens having dimensions 3 × 1 × 10 mm^3^ were mounted on the stubs.

#### 3.3.3. Thermal Properties

The elastocaloric performance strongly depends on the thermal properties of caloric materials. Therefore, the thermal diffusivity (*α*), thermal conductivity (*λ*), and specific heat capacity (*c_p_*) of the NR nanocomposites were determined at 20 °C through LFA by means of a Netzsch LFA 447 instrument (NETZSCH-Gerätebau GmbH, Selb, Germany). Cylindrical samples having a diameter of 12.7 mm and a thickness of 1 mm were used. Five pulses were performed for each specimen. The Cowan method with numerical pulse correction was employed to compute the thermal diffusivity. The reference material Pyroceram 9606 was used for the heat capacity determination, according to the standard ASTM-E 1461. The thermal conductivity was calculated according to Equation (4):

(4) $$\lambda =\alpha \cdot c_{p}\cdot \rho$$

where *ρ* is the density of the sample determined by Archimedes’ method, while *α* and *c_p_* are the results provided by the LFA measurements. For each NR formulation, three specimens were tested.

#### 3.3.4. Mechanical Properties

According to the ASTM D2240 standard, Shore A hardness was measured at 25 °C using a Hilderbrand Durometer (Prüf-und Meßtechnik GmbH, Wendlingen am Neckar, Germany). Square specimens 15 mm wide and 3 mm thick were tested, after pressing an indenter against the specimen for a time equal to 10 s. The load level was set at 488 g. Five specimens were tested for each formulation.

Quasi-static uniaxial tensile tests were performed to investigate the effect of nanoclay type and concentration on the mechanical properties of the NR nanocomposites. The tests were carried out in accordance with ASTM D412 standard, by using a universal testing machine Instron 5969 equipped with a 1 kN load cell and operating at a crosshead speed of 500 mm/min. The strain was measured with a resistance extensometer Instron 2603 having a gauge length of 25 mm. The stress corresponding to strain levels of 200% and 400% was determined (σ_200%,_ σ_400%_), together with the stress at break (σ_b_), the corresponding strain (ε_b_), and the specific tensile energy to break (E_b_), evaluated by integrating the stress–strain curve of the samples. The elastic modulus (E_100–200_) was measured as the slope of the stress–strain curve in the strain region 100–200%. Five specimens were tested for each composition.

From a mechanical point of view, the use of natural rubber in a solid-state cooler entails two implications. First, NR must undergo cyclic deformation, and second, it must be maintained in a stretched state for a specific period to promote heat exchange with the environment. Therefore, special attention is to be given to the Mullins effect, i.e., a softening that occurs in rubber (both neat and filled ones) during the first deformation, and the stress relaxation behavior of NR. It has been documented in the literature that the addition of filler to natural rubber results in a more pronounced Mullins effect [33,42,43]. To ensure that the mechanical properties remain constant over long service periods and to mitigate this effect, the rubber should be pre-stretched a certain number of times prior to use until the mechanical response has stabilized. The Mullins effect may also affect the elastocaloric properties during the initial cycles, but once the rubber is mechanically stabilized, the elastocaloric performance stabilizes as well. Therefore, to investigate the influence of nanoclay addition on the Mullins effect in the nanocomposites, uniaxial cyclic tensile tests were conducted using a universal testing machine Instron 5969 equipped with a 1 kN load cell. Rectangular specimens (25 × 10 × 3 mm^3^) were cyclically deformed 10 times up to 400% and at a crosshead speed of 125 mm/min. The strain was measured with a resistance extensometer Instron 2603 (gauge length of 25 mm). The strain level selected corresponds to the deformation used to evaluate the elastocaloric performance of the nanocomposites, and derives from the optimization performed in our previous paper on this NR system [10]. For each cycle, the energy dissipated and the stress at 400% of deformation (σ_M400_) were recorded. One specimen per composition was tested. Stress relaxation tests were carried out on an Instron 5969 equipped with a 1 kN load cell in uniaxial tension mode at 25 °C. Rectangular specimens having 1 mm thickness and 15 mm width were used, setting a gauge length of 30 mm. The specimens were deformed up to 400% at a crosshead speed of 500 mm/min and then maintained in the stretched state for 60 s (the same conditions applied in the elastocaloric tests). The stress sustained by the specimens was thus recorded as a function of time. Dividing the time-dependent stress by the applied constant strain, the trend of the relaxation modulus as a function of time was obtained. To compare the degree of stress relaxation, the percentage of stress relaxation (SR) was calculated according to Equation (5):

(5) $$SR\;\left(\%\right)=\frac{E_{0}-E_{60}}{E_{0}}\cdot 100$$

where *E*_0_ is the maximum relaxation modulus, attained when 400% of deformation is achieved, and *E*_60_ is the relaxation modulus after 60 s. To mitigate the influence of the Mullins effect, the specimens were pre-stretched three times before investigating the stress relaxation behavior. Three specimens per composition were tested.

All mechanical properties are reported as mean ± standard deviation.

#### 3.3.5. Evaluation of the Elastocaloric Effect

A high-speed tensile testing machine, STEP Lab XUD05 (STEP Lab S.r.l, Resana, TV, Italy), equipped with a 1 kN load cell, was used to investigate the elastocaloric performance of the produced nanocomposites. The tests were conducted on specimens with dimensions of 45 × 15 × 1 mm^3^ at a temperature of 22 °C and with a gauge length of 25 mm. Two specimens were tested for each natural rubber formulation, and prior to testing, each specimen was pre-stretched three times to minimize the influence of the Mullins effect and ensure more stable mechanical behavior. The procedure for assessing the elastocaloric properties consisted of four steps. (1) The specimens were deformed to 400% at a crosshead speed of 0.5 m/s. This strain level was selected because our previous research indicated it yields optimal elastocaloric performance [10]. (2) The specimens were held in the deformed state for 60 s to promote heat exchange with the environment. In this work, the cooling of rubber occurred by forced convection using a small fan (power = 2.5 W) positioned at a distance of 50 cm from the samples. (3) The specimens were unloaded at a crosshead speed of 0.5 m/s. (4) A relaxation period of 60 s was allowed for the materials to return to ambient temperature. For each formulation, two specimens were tested. Regarding the selected deformation rate, a preliminary study demonstrated that 0.5 m/s guarantees an adiabatic loading/unloading process. An infrared thermal imaging camera IRtech Fotric 348A (E Instrument Group s.r.l., Lesmo, MB, Italy), having a resolution of 0.1 °C, was used to record the surface temperature variation of the specimens both during the stretching and retraction phases. The camera was placed 50 cm from the samples, and the acquisition frequency was set at 16 fps. The emissivity parameter used was 0.9. AnalyzIR software Version 5.0.7.117 (E Instrument Group s.r.l., Lesmo, MB, Italy) was adopted to acquire punctual temperature values along the specimen’s longitudinal axis. The temperature difference between the core and the surface of the specimens was considered negligible, given the low thickness of the samples. For each specimen, the rise in temperature during the stretching phase (ΔT_heat_) and the drop in temperature during the retraction stage (ΔT_cool_) were recorded. Additionally, the characteristic time for temperature dampening (τ_c_), the heat absorbed from the surroundings per unit volume of rubber (Q_ab_), and the work required to deform the sample per unit volume of rubber (W) were calculated. To compare the cooling performance of the nanocomposites, the material coefficient of performance (COP_mat_) was also determined. The methodology adopted to calculate these parameters is the same as our previous work [10] to which the reader is referred for further details.

## 4. Conclusions

In this work, the influence of nanoclay addition, both natural (MMT) and organo-modified (O-MMT), on the elastocaloric performance of natural rubber (NR) was investigated. XRD and SEM analyses revealed a good degree of intercalation/exfoliation of the organo-modified nanoclays within the NR matrix, whereas natural MMT exhibited a strongly aggregated morphology. These morphological features translated into a more pronounced mechanical stiffening and reinforcement, associated with a more evident Mullins effect in NR/O-MMT nanocomposites.

Despite the slight increase in crosslinking density of NR induced by the presence of O-MMT, which tended to reduce the elastocaloric efficiency in unfilled NR, the NR/O-MMT samples displayed a remarkable enhancement in elastocaloric performance. In particular, organoclay-filled NR nanocomposites demonstrated up to a ~45% increase in the heat extracted per refrigeration cycle with respect to unfilled NR. More specifically, the heat absorbed from the environment increased from 11 J/cm^3^ (in the case of unfilled NR) to 16 J/cm^3^ (with an O-MMT amount of 5 phr). This value resulted in a higher cooling capacity of NR-based systems than that reported in the literature, for which a heat extracted per refrigeration cycle of 5 to 12 J/cm^3^ was determined [14,17]. The observed improvement was ascribed to a synergistic combination of enhanced strain-induced crystallization, improved macromolecular structural homogeneity, and potential strain amplification effects promoted by the organoclays. In contrast, the incorporation of natural MMT yielded only marginal improvements in both mechanical and elastocaloric properties, primarily due to poor dispersion and limited nanofiller-matrix interaction. Despite the better cooling capacity of NR/O-MMT samples, the COP_mat_ was found to be comparable in the two series of nanocomposites and ranged between 2 and 3. This was due to the greater work of deformation found in O-MMT nanocomposites, ascribed to their higher stiffness.

Overall, these findings highlighted that the selection of a proper nanofiller could play a fundamental role in enhancing the elastocaloric properties of NR. In particular, the compatibility between the rubber and the filler resulted in playing a key role in their mechanical and cooling performance. Further efforts will be devoted in the future to investigate if the better cooling capacity of NR/O-MMT samples could be attributed to a higher crystallinity developed in these systems during stretching with respect to NR/MMT samples. Specifically, the contribution of the single effects- SIC and thermoelastic effect- to the overall elastocaloric performance will be evaluated. In addition, the effect of filler functionalization on the elastocaloric performance of NR will also be deepened. Understanding the factors that enhance the cooling capacity of NR is the first step to developing solid-state cooling technologies that can compete with traditional refrigeration systems.

## Figures and Tables

**Table 1:** Density and crosslinking density of the unfilled NR and the relative nanocomposites.

Sample	Density (g/cm^3^)	Crosslinking Density (mol·10^−4^/cm^3^)
NR	0.958 ± 0.006	2.94 ± 0.21
NR_1MMT	0.960 ± 0.003	3.02 ± 0.01
NR_3MMsT	0.973 ± 0.001	3.04 ± 0.09
NR_5MMT	0.981 ± 0.005	2.94 ± 0.16
NR_1O-MMT	0.959 ± 0.001	3.32 ± 0.02
NR_3O-MMT	0.966 ± 0.001	3.73 ± 0.03
NR_5O-MMT	0.971 ± 0.003	3.68 ± 0.04

**Table 2:** Interlayer spacing for the produced nanocomposites calculated using Bragg’s law.

Sample	d_001_ Spacing [Å]
MMT	11.40
NR_5MMT	12.68
O-MMT	26.08
NR_5O-MMT	>51.70

**Table 3:** Specific heat capacity, thermal conductivity, and thermal diffusivity of neat NR and the relative nanocomposites from LFA.

Sample	Specific Heat Capacity (J/g·K)	Thermal Diffusivity (mm^2^/s)	Thermal Conductivity (W/m·K)
NR	1.70 ± 0.05	0.081 ± 0.001	0.132 ± 0.003
NR_1MMT	1.87 ± 0.08	0.085 ± 0.001	0.153 ± 0.006
NR_3MMT	1.82 ± 0.03	0.086 ± 0.001	0.151 ± 0.002
NR_5MMT	1.75 ± 0.03	0.088 ± 0.001	0.150 ± 0.003
NR_1O-MMT	1.82 ± 0.05	0.081 ± 0.002	0.141 ± 0.001
NR_3O-MMT	1.82 ± 0.04	0.081 ± 0.001	0.144 ± 0.002
NR_5O-MMT	1.75 ± 0.02	0.084 ± 0.001	0.142 ± 0.004

**Table 4:** Results of quasi-static tensile tests and Shore A hardness on the unfilled NR and the prepared nanocomposites.

Sample	E_100–200_ (MPa)	σ_200%_ (MPa)	σ_400%_ (MPa)	σ_b_ (MPa)	ε_b_ (mm/mm)	E_b_ (J/cm^3^)	Shore A
NR	0.35 ± 0.08	1.08 ± 0.13	2.10 ± 0.23	21.28 ± 3.73	730 ± 14	32.2 ± 6.4	38.0 ± 0.8
NR_1MMT	0.37 ± 0.02	1.12 ± 0.04	2.11 ± 0.15	19.45 ± 4.97	701 ± 58	28.5 ± 9.9	40.4 ± 0.5
NR_3MMT	0.42 ± 0.04	1.22 ± 0.10	2.48 ± 0.29	19.55 ± 3.05	686 ± 32	29.2 ± 6.8	40.7 ± 1.0
NR_5MMT	0.42 ± 0.06	1.20 ± 0.03	2.56 ± 0.07	18.96 ± 3.34	698 ± 30	30.2 ± 6.1	41.6 ± 0.4
NR_1O-MMT	0.44 ± 0.02	1.19 ± 0.07	2.62 ± 0.33	26.08 ± 2.94	754 ± 41	44.7 ± 12.1	42.0 ± 0.2
NR_3O-MMT	0.57 ± 0.09	1.54 ± 0.19	4.22 ± 0.46	25.39 ± 0.58	653 ± 34	42.6 ± 3.3	45.9 ± 0.2
NR_5O-MMT	0.77 ± 0.20	1.91 ± 0.33	5.36 ± 0.94	20.05 ± 3.55	630 ± 72	35.6 ± 10.9	48.0 ± 0.5

**Table 5:** Mechanisms involved in the temperature variations of the samples during the stretching and retraction phase.

Mechanism	Stretching Phase	Retraction Phase
Thermoelastic effect	↑	↓
SIC	↑	\
Internal friction macromolecules-macromolecules and macromolecules-nanoclays	↑	↑
Melting of the crystallites	\	↓

**Table 6:** Characteristic times of temperature dampening (τ_c_) calculated in the stretching and retraction phase of the prepared samples.

Sample *	τ_c,stretching_ (s)	τ_c,retraction_ (s)
NR natural convection	22.3 ± 4.6	54.1 ± 2.3
NR forced convection	13.4 ± 3.4	18.8 ± 2.2
NR_1MMT	5.7 ± 0.5	17.3 ± 0.8
NR_3MMT	6.0 ± 0.7	17.7 ± 0.1
NR_5MMT	7.5 ± 3.9	20.2 ± 2.0
NR_1O-MMT	8.8 ± 0.8	19.8 ± 0.4
NR_3O-MMT	6.7 ± 0.5	15.4 ± 0.6
NR_5O-MMT	4.8 ± 1.7	16.8 ± 0.2

* All the nanocomposite samples were tested in forced convection regime.

**Table 7:** Nanoclays used in this study. Information taken from the producers [26,37].

Trade Name	Code	Organic Modifier	Density (g/cm^3^)	d_001_ Spacing (nm)
Cloisite^®^ Na^+^	MMT	None	2.86	1.17
Cloisite^®^ 20A	O-MMT	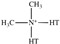	1.80	2.70

Where T is tallow (~65% C18; ~30% C16; ~5% C14) and HT is hydrogenated tallow anion: chloride.

**Table 8:** Composition in phr and nomenclature of the produced NR compounds (phr = parts per hundred rubber).

Sample	SMR 10 (phr)	Sulfur (phr)	ZnO (phr)	Stearic Acid (phr)	ZDBC (phr)	MMT (phr)	O-MMT (phr)
NR	100.0	1.5	5.0	2.0	0.7	-	-
NR_1MMT	100.0	1.5	5.0	2.0	0.7	1.0	-
NR_3MMT	100.0	1.5	5.0	2.0	0.7	3.0	-
NR_5MMT	100.0	1.5	5.0	2.0	0.7	5.0	-
NR_1O-MMT	100.0	1.5	5.0	2.0	0.7	-	1.0
NR_3O-MMT	100.0	1.5	5.0	2.0	0.7	-	3.0
NR_5O-MMT	100.0	1.5	5.0	2.0	0.7	-	5.0

## Data Availability

The original contributions presented in the study are included in the article; further inquiries can be directed to the corresponding author.

## Abbreviations

The following abbreviations are used in this manuscript:

NR	Natural rubber
ACs	Air conditioners
SIC	Strain-induced crystallization
MMT	Montmorillonite
COP	Coefficient of performance
O-MMT	Organo-modified montmorillonite
OM	Organo-modified
XRD	X-ray diffraction
LFA	Laser flash analysis
DSC	Differential scanning calorimetry
